# Evaluation in Swine of a Recombinant African Swine Fever Virus Lacking the MGF-360-1L Gene

**DOI:** 10.3390/v12101193

**Published:** 2020-10-20

**Authors:** Elizabeth Ramirez-Medina, Elizabeth A. Vuono, Ayushi Rai, Sarah Pruitt, Ediane Silva, Lauro Velazquez-Salinas, James Zhu, Douglas P. Gladue, Manuel V. Borca

**Affiliations:** 1 Plum Island Animal Disease Center, ARS, USDA, Greenport, NY 11944, USA; Elizabeth.Ramirez@usda.gov (E.R.-M.); Elizabeth.Vuono@usda.gov (E.A.V.); Ayushi.Rai@usda.gov (A.R.); Sarah.Pruitt@usda.gov (S.P.); Ediane.Silva@usda.gov (E.S.); Lauro.Velazquez@usda.gov (L.V.-S.); James.Zhu@usda.gov (J.Z.); 2 Department of Pathobiology and Veterinary Science, University of Connecticut, Storrs, CT 06269, USA; 3 Department of Pathobiology and Population Medicine, Mississippi State University, P.O. Box 6100, Mississippi, MS 39762, USA; 4 Oak Ridge Institute for Science and Education (ORISE), Oak Ridge, TN 37830, USA; 5 Department of Anatomy and Physiology, Kansas State University, Manhattan, KS 66506, USA

**Keywords:** ASFV, ASF, African swine fever virus, MGF360-1L

## Abstract

The African swine fever (ASF) pandemic is currently affecting pigs throughout Eurasia, resulting in significant swine production losses. The causative agent, ASF virus (ASFV), is a large, structurally complex virus with a genome encoding more than 160 genes. The function of most of those genes remains unknown. Here, we presented the previously uncharacterized ASFV gene MGF360-1L, the first gene in the genome. The kinetic studies of virus RNA transcription demonstrated that the MGF360-1L gene was transcribed as a late virus protein. The essentiality of MGF360-1L to virus replication was evaluated by developing a recombinant ASFV lacking the gene (ASFV-G-ΔMGF360-1L). In primary swine macrophage cell cultures, ASFV-G-ΔMGF360-1L showed similar replication kinetics as the parental highly virulent field isolate Georgia2007 (ASFV-G). Domestic pigs experimentally infected with ASFV-G-ΔMGF360-1L presented with a clinical disease indistinguishable from that caused by ASFV-G, demonstrating that MGF360-1L was not involved in virulence in swine, the natural host of ASFV.

## 1. Introduction

African swine fever (ASF) is a disease of domestic and wild swine that produces a spectrum of disease, from sub-clinical to highly lethal, depending on the acting virus strain [1]. The causative agent, ASF virus (ASFV), is a large, highly structured, enveloped DNA virus with a double-stranded DNA genome (180–190 kilobase pairs) encoding for at least 160 open reading frames (ORFs) [1]. The identification of viral proteins involved in virus replication and virus virulence in swine is critical to developing novel countermeasures to control the disease. However, the role of most of these ORFs has been predicted using functional genomics without experimental characterization, limiting application to the development of therapeutics.

Although historically restricted to sub-Saharan countries and Sardinia (Italy), the disease was detected in 2007 in the Republic of Georgia and has since expanded into Eastern Europe, China, and South Asia [2]. There are no commercially available vaccines for ASF, so disease outbreaks are usually quelled by removing infected animals. The rapid spread of ASF, combined with its high lethality, makes it a significant threat to global protein availability [2].

Experimental vaccines have been developed that effectively produce protection against the current strain circulating in Europe and Asia. These experimental vaccines are recombinant live attenuated viruses derived from the virus isolate that initiated the outbreak in the Republic of Georgia in 2007. These recombinant viruses are attenuated by deleting one or more genes from the field isolate genome by genetic manipulation [3,4,5,6,7,8,9]. Interestingly, only a few virus genes have been successfully deleted from the ASFV Georgia genome (e.g., TK, NL, CD2, MGF360-16R, L83L, C962R, X69R) [10,11,12,13,14,15], while a smaller number of genes have been shown to be essential for virus replication (e.g., EP152R, p. 30, p. 54, p. 72) [16,17,18,19]. Understanding the role of individual genes in virus virulence is a critical step in the development of novel ASF vaccines. However, the lack of experimental information limits the understanding of gene function and the possibility of using that knowledge to develop novel countermeasures to control the disease.

ASFV Georgia (ASFV-G) contains 16 genes within the multigene family 360 (MGF360) located on both ends of the ASFV-G genome—thirteen genes in the left terminal repeat region (MGF360-1L, 2L, 3L, 4L, 6L, 8L, 9L, 10L, 11L, 12L, 13L, 14L, and 15L) and three genes in the right terminal repeat region (MGF360-16R, 19R, and 21R). There are currently 19 ORFs that are considered paralogs that belong to MGF360, with ASFV-G missing genes annotated as MGF360-1.5L, 5L, 22R (these three members of the MGF360 family have been found in very few sequenced ASFV genomes). Recently, MGF360- 17R, 18R, and 20R are determined not to be members of the MGF360, as they were initially misannotated [20]. The lengths of all MGF360 paralogs are highly conserved, approximately 960–1100 bp in length for all known family members in all sequenced isolates. The MGF360 gene family contains several examples of ortholog fusions and truncations and contains cross-MGF-series fusions [20]. It has been shown that the deletion of some of the MGF360 genes along with others of the MGF505 gene family on the left arm of the genome produces no attenuation of highly virulent isolate Georgia 2010 [11,21]. A recombinant virus with a large deletion of the MGF360 family along with the MGF505 family does not induce circulating interferon when compared to the parental virus, yet cell culture interferon could inhibit non-virulent ASFV with a deletion of similar MGF genes [22]. Besides, it has been described that ASFV replication in *Ornithodoros porcinus* ticks, a vector of ASFV, is dependent on the presence of specific MGF360 genes [23]. However, only a small number of reports have studied the role of any individual MGF genes [11]. Currently, MGF360-1L has not been studied individually or as part of any larger deletions through virus adaptation or recombination studies.

In this report, we studied the role of the previously uncharacterized ASFV gene MGF360-1L, the first gene encoded in the genome of ASFV-G. In this study, a recombinant ASFV lacking the MGF360-1L gene was constructed (ASFV-G-ΔMGF360-1L) and assessed for its ability to replicate in vitro and in vivo. In addition, the experimental infection of domestic pigs with ASFV-G-ΔMGF360-1L demonstrated that MGF360-1L was not essential for virus virulence.

## 2. Materials and Methods

### 2.1. Viruses and Pimary Swine Macrophage Cultures 

Primary swine macrophage cell cultures were prepared from defibrinated swine blood, as previously described [24]. Briefly, heparin-treated swine blood was incubated at 37 °C for 1 h to allow sedimentation of the erythrocyte fraction. Mononuclear leukocytes were separated by flotation over a Ficoll-Paque (Pharmacia, Piscataway, NJ, USA) density gradient (specific gravity, 1.079). The monocyte/macrophage cell fraction was cultured in plastic Primaria (Falcon; Becton Dickinson Labware, Franklin Lakes, NJ, USA) tissue culture flasks containing macrophage media, composed of RPMI 1640 Medium (Life Technologies, Grand Island, NY, USA) with 30% L929 supernatant and 20% fetal bovine serum (HI-FBS, Thermo Scientific, Waltham, MA, USA) for 48 h at 37 °C under 5% CO_2_.

Adherent cells were detached from the plastic by using 10 mM EDTA in phosphate-buffered saline (PBS) and were then reseeded into Primaria T25, 6- or 96-well dishes at a density of 5 × 10^6^ cells per mL for use in assays 24 h later. ASFV Georgia (ASFV-G) was a field isolate, kindly provided by Dr. Nino Vepkhvadze, from the Laboratory of the Ministry of Agriculture (LMA) in Tbilisi, Republic of Georgia.

### 2.2. Virus Growth Curves

Comparative growth curves between ASFV-G-ΔMGF360-1L and parental ASFV-G were performed in primary swine macrophage cell cultures. Preformed monolayers were prepared in 24-well plates and infected at an MOI (Multiplicity of Infection) of 0.1 (based on hemoadsorbing doses, HAD50, previously determined in primary swine macrophage cell cultures). After 1 h of adsorption at 37 °C under 5% CO2, the inoculum was removed, and the cells were rinsed two times with PBS. The monolayers were then rinsed with macrophage media and incubated for 2, 24, 48, 72, and 96 h at 37 °C under 5% CO2. At appropriate times post-infection, the cells were frozen at ≤−70 °C, and the thawed lysates were used to determine titers by HAD50/mL in primary swine macrophage cell cultures. All samples were run simultaneously to avoid inter-assay variability. Virus titration was performed on primary swine macrophage cell cultures in 96-well plates. Virus dilutions and cultures were performed using macrophage medium. The presence of the virus was assessed by hemadsorption (HA), and the virus titers were calculated, as previously described [25].

### 2.3. Construction of the Recombinant Viruses

**Figure 1:**
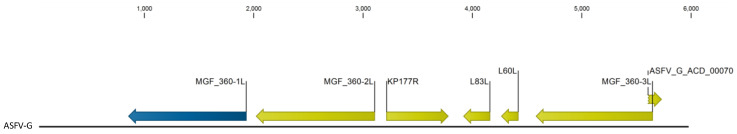
Schematic representation of the MGF360-1L ORF (blue) in the ASFV-G genome, showing adjacent open reading frames (yellow). ORF, open reading frame.

Recombinant ASFV-G-ΔMGF360-1L was generated by homologous recombination between the parental ASFV genome and a recombination transfer vector following previously described procedures [21]. The recombinant transfer vector (p72mCherryΔMGF360-1L) contained flanking genomic regions: the left arm was located between genomic positions 1 and 861, and the right arm was located between genomic positions 1935 and 2935, and there was a reporter gene cassette containing the mCherry fluorescent protein (mCherry) gene under the control of the ASFV p72 late gene promoter, as previously described [21]. The recombinant transfer vector was obtained by DNA synthesis (Epoch Life Sciences, Sugar Land, TX, USA). This construction created a 1073-nucleotide deletion between nucleotide positions 862 and 1934, deleting most of the ORF sequence for MGF360-1L with the coding region for the last 10 nucleotides of the C-terminus remaining (Figure 1). Macrophage cell cultures were infected with ASFV-G and transfected with p72mCherryΔMGF360-1L. ASFV-G-ΔMGF360-1L was obtained as a pure population after ten successive limiting dilution purification steps in swine macrophage cell cultures. ASFV-G-ΔMGF360-1L stocks were obtained after a further amplification of the virus from the last round of purification.

ASFV DNA was extracted from infected cells, and a full-length sequence was obtained using next-generation sequencing (NGS), as described previously [21], using an Illumina NextSeq500 sequencer, using standard sequencing protocols. The analysis of the sequence was done using CLC Genomics Workbench software version 20 (QIAGEN, Hilden, Germany). 

### 2.4. Animal Experiments

ASFV-G-ΔMGF360-1L was assessed for its virulence phenotype relative to the parental ASFV-G virus using 80–90 pound Yorkshire crossbred female swine. Five pigs were inoculated intramuscularly (IM) with 10^2^ HAD_50_ of ASFV-G-ΔMGF360-1L and compared with a group of pigs inoculated with similar doses of ASFV-G. Clinical signs (anorexia, depression, fever, purple skin discoloration, staggering gait, diarrhea, and cough) and changes in rectal body temperature were recorded daily throughout the experiment (including the 5 days acclimation period). The original schedule considered blood sampling times on days 4, 7, 11, 14, 21, and 28 post-infection.

### 2.5. Ethics Statement

Animal experiments were performed under biosafety level 3AG conditions in the animal facilities at Plum Island Animal Disease Center (PIADC). All experimental procedures were carried out in compliance with the Animal Welfare Act (AWA), the 2011 Guide for Care and Use of Laboratory Animals, the 2002 PHS Policy for the Humane Care and Use of Laboratory Animals, and the U.S. Government Principles for Utilization and Care of Vertebrate Animals Used in Testing, Research, and Training (IRAC 1985), as well as specific animal protocols reviewed and approved by the PIADC Institutional Animal Care and Use Committee of the U.S. Departments of Agriculture and Homeland Security (protocol number 225.04-16-R, 09-07-16, approved on 9/10/19). 

## 3. Results and Discussion

### 3.1. MGF360-1L Gene is Conserved in Most ASFV Isolates

ASFV ORF MGF360-1L is the first gene encoded in the ASFV genome and is located on the negative strand of the ASFV-G genome between positions 852 and 1934. MGF360-1L is present in the most sequenced isolates of ASFV, the exception being ASFV E75, which contains an MGF member consisting of a fusion of ORFs—MGF360-1L and MGF360-2L. MGF360-1L is also absent from the genomes of Mkuzi_1975, Ken06_Bus, and Malawi isolates. In the Malawi isolate, the genomic region for MGF360-1L has the gene MGF360-21R, a protein unique to the Malawai isolate [20]. InterPro sequence analysis [26] of MGF360-1L revealed that amino acids 99-309 are those that distinguish MGF360-1L as a member of the MGF360 family. Furthermore, MGF360-1L is not detected in the proteome of ASFV viral particles, which would be expected of genes involved in viral DNA replication [27]. These predicted domains and locations in the genome are shown in Figure 1.

**Figure 2:**
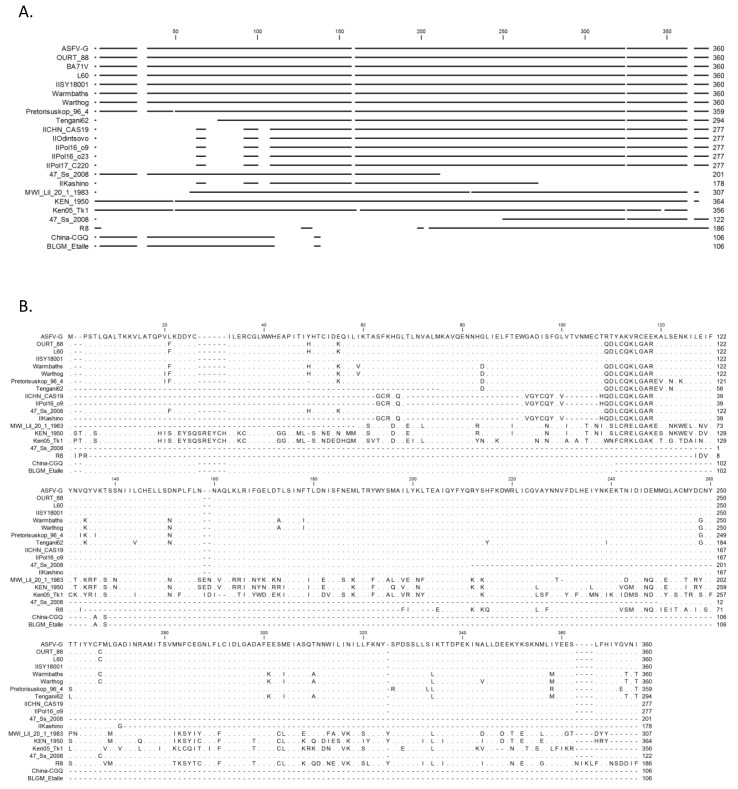
Multiple sequence alignment of the indicated ASFV isolates of viral protein MGF360-1L. (**A**) An overview of matching areas of the protein. (**B**) Amino acid level alignment with matching residues are represented as dots and missing residues as dashes. ASFV, African swine fever virus.

Multiple amino acid sequence alignments across all published isolates of ASFV that contain MGF360-1L were extracted using the Viral Bioinformatics Research Centers Viral Orthologous Clusters program and analyzed using CLC Genomics Workbench, revealing diversity in the MGF360-1L proteins across ASFV genomes. MGF360-1L proteins vary in length, between 122 and 160 amino acids. Overall alignment (Figure 2A) revealed that there is no conserved region within the MGF360-1L protein across all virus isolates, which is not a surprise as some isolates of ASFV do not have MGF360-1L. When compared to ASFV-G, an isolate containing one of the largest MGF360-1L proteins, some isolates contain a smaller MGF360-1L that lacks the N-terminus region, the C-terminus region, or the middle region of the protein. Further analysis at the amino acid level revealed a high degree of conservation across isolates harboring similar areas of MGF360-1L, with the exception of the Kenya, Malawi, and R8 isolates, which have a higher degree of conservation with each other. In addition, there is a region at amino acids 108-121, which appears to have two different groups of sequences, one group being very similar to ASFV-G from both recent outbreak isolates and historical isolates (L60), and a separate group being similar to that of the OURT/88 isolate.

Previously deposited microarray data from a previous study [28] was used to determine the transcriptional activity of the MGF360-1L gene during the infectious cycle kinetics of RNA transcription in primary swine macrophages infected with ASFV-G. We determined that the transcription of MGF360-1L occurs early with RNA that hybridizes to the microarray starting at 3 h post-infection, with increasing amounts of RNA throughout the remaining duration of infection, and these expression kinetics are similar to ASFV early protein p30 (CP204L) that has been previously reported using this microarray data [3,11].

### 3.2. Development of the ASFV-G-ΔMGF360-1LGgene Deletion Mutant

**Figure 3:**
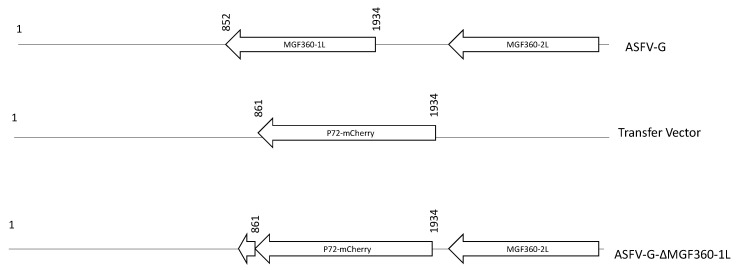
Schematic for the development of ASFV-G-ΔMGF360-1L. The transfer vector contains the p72 promoter and a mCherry cassette; the flanking left and right arms are designed to have flanking ends to both sides of the deletion/insertion cassette. The resulting ASFV-G-ΔMGF360-1L with the cassette inserted is shown on the bottom with the residual 10 amino acids of MGF360-1L, which are unlikely to be transcribed, as indicated by the shortened arrow. The nucleotide positions shown are relative to the parental virus.

To determine the function, if any, of the MGF360-1L gene during in vitro virus replication, a recombinant ASFV was developed (ASFV-G-ΔMGF360-1L) using the highly virulent isolate ASFV Georgia 2007 (ASFV-G) as a template (Figure 3). ASFV-G-ΔMGF360-1L genomic modifications resulted in the partial deletion of the MGF360-1L ORF, leaving the C-terminal 10 bp.

Next-generation sequencing (NGS) was used to assess both the accuracy of the genetic modifications introduced during recombination and the conservation of the genomic integrity of the rest of the virus genome. Full-length genomic comparison between ASFV-G-ΔMGF360-1L and parental ASFV-G demonstrated a deletion of 1073 nucleotides and the insertion of a 1294-nucleotide construct corresponding to the p72-mCherry cassette sequence. No additional significant differences are observed between these two virus genomes. This confirmed that ASFV-G-ΔMGF360-1L does not acquire additional mutations during the process of homologous recombination or purification steps. Besides, NGS data indicated the absence of any residual parental ASFV-G genome as a contaminant of the ASFV-G-ΔMGF360-1L virus stock.

### 3.3. Assessment of the Ability of ASFV-G-ΔMGF360-1L to Replicate in Swine Macrophages

**Figure 4:**
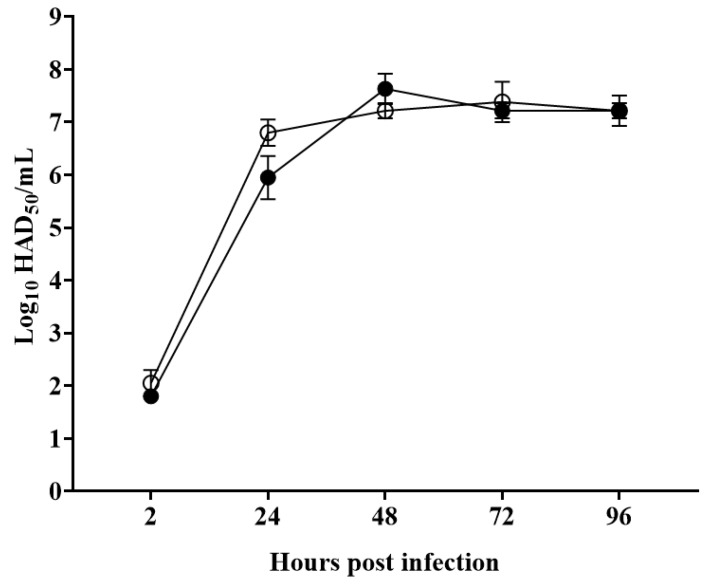
In vitro growth kinetics in primary swine macrophage cell cultures for ASFV-G-ΔMGF360-1L. Macrophages cultures were infected (MOI = 0.01) with either ASFV-G-ΔMGF360-1L (filled circles) or parental ASFV-G (empty circles) viruses. Samples were taken from three independent experiments at the indicated time points and titrated. Data represent means and standard deviations. Sensitivity using this methodology for detecting virus: ≥log_10_ 1.8 HAD_50_/mL. (Median Hemeadsorbing infectious dose/milliliter) No significant differences in viral yields between viruses are observed at any time point tested determined using the Holm–Sidak method (α = 0.05), without assuming a consistent standard deviation. All calculations were conducted using the software Graphpad Prism version 8.

Several members of the ASFV multigene families have been shown to be involved in the process of virus replication in its main target cell type, the macrophage [23,29]. To evaluate the potential role of MGF360-1L in virus replication, the in vitro growth kinetics of ASFV-G-ΔMGF360-1L was studied in swine macrophage cultures and compared with that of the parental ASFV-G in a multistep growth curve. Results demonstrated that ASFV-G-ΔMGF360-1L presents an almost indistinguishable growth kinetic when compared to the parental ASFV-G (Figure 4). Thus, the deletion of the MGF360-1L gene does not significantly affect the ability of ASFV-G to replicate in swine macrophages. This was somewhat unexpected, considering the deletion of most of the MGF genes studied affects the ability of the recombinant virus to replicate in macrophages. Members of MGF360 have been shown to be involved in the process of virus replication in both swine macrophages, the main target cell during the infection in swine, and in cells from ticks, the alternative natural host [7,23]. However, it is possible that the MGF360-1L function can be replaced by many of the MGF genes remaining in the ASFV-G-ΔMGF360-1L genome. Previous studies have demonstrated that the deletion of specific MGF360 genes 13L, 14L, and 16R does not significantly affect virus replication in swine macrophages [11,21]. Similarly, the deletion of MGF360-1L alone appears to not affect ASFV replication in swine macrophages. However, it is possible that the MGF360-1L function can be replaced by other MGF genes present in the ASFV-G-ΔMGF360-1L genome.

### 3.4. Assessing the Role of the MGF360-1L Gene in Virulence During Swine Infection

Although replication of ASFV-G-ΔMGF360-1L in primary swine macrophages cultures showed no significant differences, it was important to determine if the recombinant virus efficiently replicates in vivo and produces disease as efficiently as the parental ASFV-G. Several members of MGF360 and MGF505 are directly involved in virus virulence during infection in domestic swine [7,30,31]. The absence of several genes within the MGF360/505 in naturally attenuated isolates (as NHV and OUT88/3) has been associated with a reduction of virulence in swine [32]. In addition, losing members of these multigene families during adaptation of virulent field isolates (as BA71, Lisbon60, and Georgia 2010) to established cell lines has been linked to the progressive decrease of virulence in swine [29,33,34]. Simultaneous experimental deletion or insertion of several members of the MGF360/505 can result in the attenuation of virulent parental viruses (i.e., Benin and Georgia) [7,30] or increased virulence of an attenuated strain (i.e., E70∆NL) [31].

**Figure 5:**
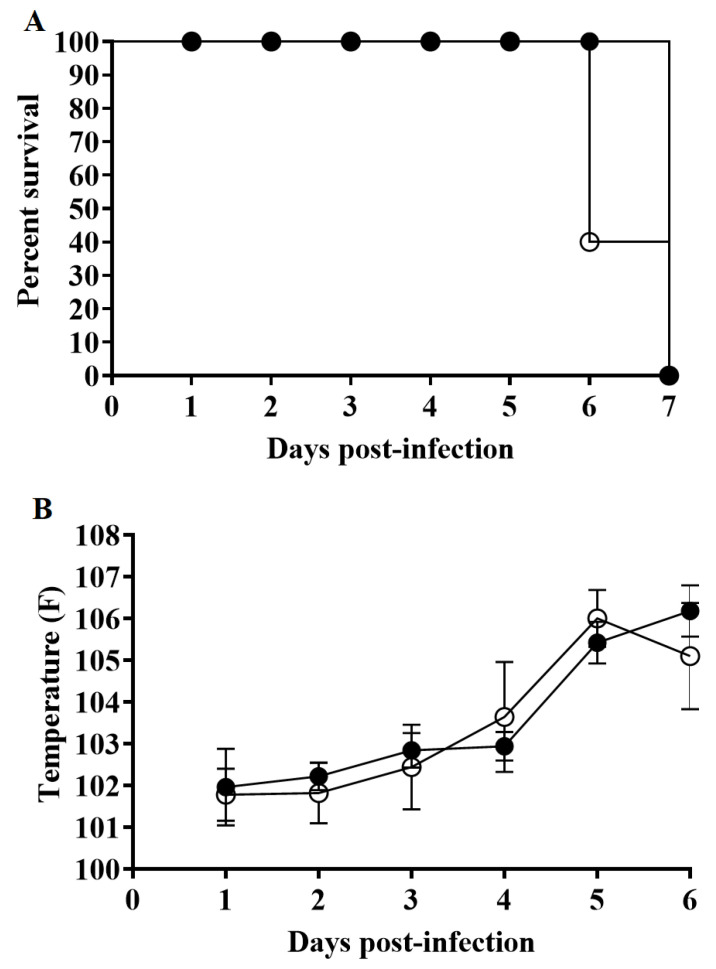
Evolution of mortality (**A**) and body temperature (**B**) in animals (5 animals/group) IM inoculated with 10^2^ HAD_50_ of either ASFV-G-ΔMGF360-1L (filled symbols) or parental ASFV-G (open symbols). No significant differences in rectal temperatures between groups of pigs are found at any sample time tested using the Holm–Sidak method (α = 0.05) without assuming a consistent standard deviation. All calculations were conducted using the software Graphpad Prism version 8.

To assess how the deletion of the MGF360-1L gene affects ASFV-G virulence, a group (*n* = 5) of 80–90-pound pigs were IM inoculated with 10^2^ HAD_50_ ASFV-G-ΔMGF360-1L and compared with a control group IM inoculated with 10^2^ HAD_50_ parental ASFV-G. Animals inoculated with virulent ASFV-G have elevated body temperature (>104 °F) by day 4–5 pi, followed by the rapid development of ASF-associated clinical acute disease (anorexia, depression, purple skin discoloration, staggering gait and diarrhea, and shivering) (Table 1 and Figure 5).

The clinical disease rapidly evolves into a severe form, with all animals euthanized in extremis by day 6–7 pi. Interestingly, animals inoculated with ASFV-G-ΔMGF360-1L present with a clinical disease almost indistinguishable from that observed in animals infected with ASFV-G. Both the time to presentation and severity of the clinical signs resemble those present in animals inoculated with the parental virus. The deletion of the MGF360-1L gene from the ASFV-G genome does not significantly alter virulence.

**Figure 6:**
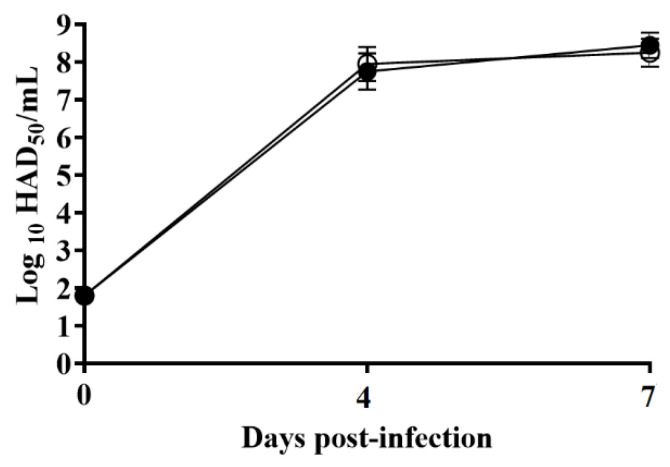
Viremia titers detected in pigs IM inoculated with 10^2^ HAD_50_ of either ASFV-G-ΔMGF360-1L (filled symbols) or ASFV-G (empty symbols). Each symbol represents the average of animal titers in each of the groups. Sensitivity of virus detection: > log_10_ 1.8 TCID_50_/mL. No significant differences in viremia values between both groups of pigs are found at any sample time tested using the Holm–Sidak method (α = 0.05) without assuming a consistent standard deviation. All calculations were conducted on the software Graphpad Prism version 8.

Viremias in animals IM inoculated with parental ASFV-G showed expected high titers (10^7^–10^8^ HAD_50_/mL) on day 4 pi, remaining high until day 7 pi, when all animals were euthanized. Similarly, animals infected with ASFV-G-ΔMGF360-1L present by day 4 with viremia values as high as those found in the ASFV-G-infected group (Figure 6).

In summary, we determined that ASFV-G-ΔMGF360-1L is a relatively conserved protein among most ASFV isolates, which is expressed as an early protein during virus replication. It is a non-essential gene since its deletion from the ASFV-G genome does not significantly alter virus replication in vitro or in vivo. Importantly, MGF360-1L is not critical for ASFV virulence in swine, as the deletion mutant ASFV-G-ΔMGF360-1L has similar pathogenesis as the parental ASFV-G. It is interesting that although a group of MGFs is involved in virulence in swine [7,29,31,32,33,34,35], individual gene deletion so far cannot be shown to reduce virulence [3,11].

To date, no reports characterizing the functionality of ASFV-G-ΔMGF360-1L have been published. A large deletion (approximately 14.5 Kb) in the left end of the Estonian isolate genome (a derivative of the ASFV Georgia 2007 isolate) is associated with decreased virulence in domestic swine [36]. Twenty-six genes, including MGF360-1L, were naturally deleted in this isolate, making it difficult to evaluate the contribution of MGF360-1L to virus virulence. From our results, it is evident that the deletion of the MGF360-1L gene by itself does not affect virus replication or disease phenotype in domestic swine. As it has been shown in many studies evaluating genes that are critical for virus replication and/or pathogenesis, the functional assessment of previously uncharacterized ASFV genes is critical to advance the development of novel experimental vaccines.

## Figures and Tables

**Table 1:** Swine survival and fever response following infection with ASFV-G-ΔMGF360-1L and parental ASFV-G.

			Fever		
Virus (10^2^ HAD_50_)	No. of Survivors/Total	Mean Time to Death (±SD ^1^)	No. of Days to Onset (±SD ^1^)	Duration No. of Days (±SD ^1^)	Maximum Daily Temp, °F (±SD ^1^)
ASFV-G-ΔMGF360-1L	0/5	7 (0)	5 (0)	2 (0)	106.18 (0.61)
ASFV-G	0/5	6.4 (0.55)	4.4 (0.55)	2 (1)	106 (0.69)

^1^ SD (Standard Deviation).
