# Erratum: Ramirez-Medina, E.; et al. Evaluation in Swine of a Recombinant African Swine Fever Virus Lacking the MGF-360-1L Gene. *Viruses* 2020, *12*, 1193

**DOI:** 10.3390/v12111307

**Published:** 2020-11-16

**Authors:** Viruses Editorial Office

**Affiliations:** MDPI AG, St. Alban-Anlage 66, 4052 Basel, Switzerland; viruses@mdpi.com

The Viruses Editorial Office wishes to notify its readers of corrections in [1].

The following references are from another paper:

1. Akiyama, B.M.; Laurence, H.M.; Massey, A.R.; Costantino, D.A.; Xie, X.; Yang, Y.; Shi, P.Y.; Nix, J.C.; Beckham, J.D.; Kieft, J.S. Zika virus produces noncoding RNAs using a multi-pseudoknot structure that confounds a cellular exonuclease. *Science* **2016**, *354*, 1148–1152.
2. Pijlman, G.P.; Funk, A.; Kondratieva, N.; Leung, J.; Torres, S.; van der Aa, L.; Liu, W.J.; Palmenberg, A.C.; Shi, P.Y.; Hall, R.A.; et al. A highly structured, nuclease-resistant, noncoding RNA produced by flaviviruses is required for pathogenicity. *Cell Host Microbe* **2008**, *4*, 579–591.
3. Chapman, E.G.; Costantino, D.A.; Rabe, J.L.; Moon, S.L.; Wilusz, J.; Nix, J.C.; Kieft, J.S. The structural basis of pathogenic subgenomic flavivirus RNA (sfRNA) production. *Science* **2014**, *344*, 307–310.
4. Chang, R.Y.; Hsu, T.W.; Chen, Y.L.; Liu, S.F.; Tsai, Y.J.; Lin, Y.T.; Chen, Y.S.; Fan, Y.H. Japanese encephalitis virus non-coding RNA inhibits activation of interferon by blocking nuclear translocation of interferon regulatory factor 3. *Vet. Microbiol.* **2013**, *166*, 11–21.
5. Chapman, E.G.; Moon, S.L.; Wilusz, J.; Kieft, J.S. RNA structures that resist degradation by Xrn1 produce a pathogenic Dengue virus RNA. *Elife* **2014**, *3*, e01892.
6. Goertz, G.P.; Fros, J.J.; Miesen, P.; Vogels, C.B.F.; van der Bent, M.L.; Geertsema, C.; Koenraadt, C.J.M.; van Rij, R.P.; van Oers, M.M.; Pijlman, G.P. Noncoding Subgenomic Flavivirus RNA Is Processed by the Mosquito RNA Interference Machinery and Determines West Nile Virus Transmission by Culex pipiens Mosquitoes. *J. Virol.* **2016**, *90*, 10145–10159.
7. Moon, S.L.; Dodd, B.J.; Brackney, D.E.; Wilusz, C.J.; Ebel, G.D.; Wilusz, J. Flavivirus sfRNA suppresses antiviral RNA interference in cultured cells and mosquitoes and directly interacts with the RNAi machinery. *Virology* **2015**, *485*, 322–329.
8. Schnettler, E.; Sterken, M.G.; Leung, J.Y.; Metz, S.W.; Geertsema, C.; Goldbach, R.W.; Vlak, J.M.; Kohl, A.; Khromykh, A.A.; Pijlman, G.P. Noncoding flavivirus RNA displays RNA interference suppressor activity in insect and Mammalian cells. *J. Virol.* **2012**, *86*, 13486–13500.
9. Schnettler, E.; Tykalova, H.; Watson, M.; Sharma, M.; Sterken, M.G.; Obbard, D.J.; Lewis, S.H.; McFarlane, M.; Bell-Sakyi, L.; Barry, G.; et al. Induction and suppression of tick cell antiviral RNAi responses by tick-borne flaviviruses. *Nucleic Acids Res.* **2014**, *42*, 9436–9446.
10. Schuessler, A.; Funk, A.; Lazear, H.M.; Cooper, D.A.; Torres, S.; Daffis, S.; Jha, B.K.; Kumagai, Y.; Takeuchi, O.; Hertzog, P.; et al. West Nile virus noncoding subgenomic RNA contributes to viral evasion of the type I interferon-mediated antiviral response. *J. Virol.* **2012**, *86*, 5708–5718.
11. Pallares, H.M.; Costa Navarro, G.S.; Villordo, S.M.; Merwaiss, F.; de Borba, L.; Gonzalez Lopez Ledesma, M.M.; Ojeda, D.S.; Henrion-Lacritick, A.; Morales, M.A.; Fabri, C.; et al. Zika Virus Subgenomic Flavivirus RNA Generation Requires Cooperativity between Duplicated RNA Structures That Are Essential for Productive Infection in Human Cells. *J. Virol.* **2020**, *94*, doi:10.1128/JVI.00343-20.
12. Liu, Y.; Liu, H.; Zou, J.; Zhang, B.; Yuan, Z. Dengue virus subgenomic RNA induces apoptosis through the Bcl-2-mediated PI3k/Akt signaling pathway. *Virology* **2014**, *448*, 15–25.
13. Weger-Lucarelli, J.; Duggal, N.K.; Bullard-Feibelman, K.; Veselinovic, M.; Romo, H.; Nguyen, C.; Ruckert, C.; Brault, A.C.; Bowen, R.A.; Stenglein, M.; et al. Development and Characterization of Recombinant Virus Generated from a New World Zika Virus Infectious Clone. *J. Virol.* **2017**, *91*, doi:10.1128/JVI.01765-16.
14. Brien, J.D.; Lazear, H.M.; Diamond, M.S. Propagation, quantification, detection, and storage of West Nile virus. *Curr. Protoc. Microbiol.* **2013**, *31*, 15D-3.
15. Luo, C.; Zuniga, J.; Edison, E.; Palla, S.; Dong, W.; Parker-Thornburg, J. Superovulation strategies for 6 commonly used mouse strains. *J. Am. Assoc. Lab. Anim. Sci.* **2011**, *50*, 471–478.
16. Rio, D.C.; Ares, M., Jr.; Hannon, G.J.; Nilsen, T.W. Purification of RNA using TRIzol (TRI reagent). *Cold Spring Harb. Protoc.* **2010**, *2010*, pdb-prot5439.
17. Lazear, H.M.; Govero, J.; Smith, A.M.; Platt, D.J.; Fernandez, E.; Miner, J.J.; Diamond, M.S. A Mouse Model of Zika Virus Pathogenesis. *Cell Host Microbe* **2016**, *19*, 720–730.
18. Gorman, M.J.; Caine, E.A.; Zaitsev, K.; Begley, M.C.; Weger-Lucarelli, J.; Uccellini, M.B.; Tripathi, S.; Morrison, J.; Yount, B.L.; Dinnon, K.H., 3rd; et al. An Immunocompetent Mouse Model of Zika Virus Infection. *Cell Host Microbe* **2018**, *23*, 672–685.e6.
19. Moon, S.L.; Anderson, J.R.; Kumagai, Y.; Wilusz, C.J.; Akira, S.; Khromykh, A.A.; Wilusz, J. A noncoding RNA produced by arthropod-borne flaviviruses inhibits the cellular exoribonuclease XRN1 and alters host mRNA stability. *RNA* **2012**, *18*, 2029–2040.
20. Manokaran, G.; Finol, E.; Wang, C.; Gunaratne, J.; Bahl, J.; Ong, E.Z.; Tan, H.C.; Sessions, O.M.; Ward, A.M.; Gubler, D.J.; et al. Dengue subgenomic RNA binds TRIM25 to inhibit interferon expression for epidemiological fitness. *Science* **2015**, *350*, 217–221.
21. Donald, C.L.; Brennan, B.; Cumberworth, S.L.; Rezelj, V.V.; Clark, J.J.; Cordeiro, M.T.; Freitas de Oliveira Franca, R.; Pena, L.J.; Wilkie, G.S.; Da Silva Filipe, A.; et al. Full Genome Sequence and sfRNA Interferon Antagonist Activity of Zika Virus from Recife, Brazil. *PLoS Negl. Trop. Dis.* **2016**, *10*, e0005048.
22. Bidet, K.; Dadlani, D.; Garcia-Blanco, M.A. G3BP1, G3BP2 and CAPRIN1 are required for translation of interferon stimulated mRNAs and are targeted by a dengue virus non-coding RNA. *PLoS Pathog.* **2014**, *10*, e1004242.
23. Baxter, V.K.; Griffin, D.E. Interferon gamma modulation of disease manifestation and the local antibody response to alphavirus encephalomyelitis. *J. Gen. Virol.* **2016**, *97*, 2908–2925.
24. Bayer, A.; Lennemann, N.J.; Ouyang, Y.; Bramley, J.C.; Morosky, S.; Marques, E.T., Jr.; Cherry, S.; Sadovsky, Y.; Coyne, C.B. Type III Interferons Produced by Human Placental Trophoblasts Confer Protection against Zika Virus Infection. *Cell Host Microbe* **2016**, *19*, 705–712.
25. Binder, G.K.; Griffin, D.E. Interferon-gamma-mediated site-specific clearance of alphavirus from CNS neurons. *Science* **2001**, *293*, 303–306.
26. Jagger, B.W.; Miner, J.J.; Cao, B.; Arora, N.; Smith, A.M.; Kovacs, A.; Mysorekar, I.U.; Coyne, C.B.; Diamond, M.S. Gestational Stage and IFN-lambda Signaling Regulate ZIKV Infection In Utero. *Cell Host Microbe* **2017**, *22*, 366–376.e3.
27. Baxter, V.K.; Griffin, D.E. Interferon-Gamma Modulation of the Local T Cell Response to Alphavirus Encephalomyelitis. *Viruses* **2020**, *12*, 113.
28. Caine, E.A.; Jagger, B.W.; Diamond, M.S. Animal Models of Zika Virus Infection during Pregnancy. *Viruses* **2018**, *10*, 598.
29. Carbaugh, D.L.; Zhou, S.; Sanders, W.; Moorman, N.J.; Swanstrom, R.; Lazear, H.M. Two genetic differences between closely-related Zika virus strains determine pathogenic outcome in mice. *J. Virol.* **2020**, *94*, doi:10.1128/JVI.00618-20.
30. Shan, C.; Muruato, A.E.; Jagger, B.W.; Richner, J.; Nunes, B.T.D.; Medeiros, D.B.A.; Xie, X.; Nunes, J.G.C.; Morabito, K.M.; Kong, W.P.; et al. A single-dose live-attenuated vaccine prevents Zika virus pregnancy transmission and testis damage. *Nat. Commun.* **2017**, *8*, 676.
31. Shan, C.; Muruato, A.E.; Nunes, B.T.D.; Luo, H.; Xie, X.; Medeiros, D.B.A.; Wakamiya, M.; Tesh, R.B.; Barrett, A.D.; Wang, T.; et al. A live-attenuated Zika virus vaccine candidate induces sterilizing immunity in mouse models. *Nat. Med.* **2017**, *23*, 763–767.
32. Zou, J.; Xie, X.; Luo, H.; Shan, C.; Muruato, A.E.; Weaver, S.C.; Wang, T.; Shi, P.Y. A single-dose plasmid-launched live-attenuated Zika vaccine induces protective immunity. *EBioMedicine* **2018**, *36*, 92–102.
33. Beck, A.; Tesh, R.B.; Wood, T.G.; Widen, S.G.; Ryman, K.D.; Barrett, A.D. Comparison of the live attenuated yellow fever vaccine 17D-204 strain to its virulent parental strain Asibi by deep sequencing. *J. Infect. Dis.* **2014**, *209*, 334–344.
34. Blaney, J.E., Jr.; Sathe, N.S.; Goddard, L.; Hanson, C.T.; Romero, T.A.; Hanley, K.A.; Murphy, B.R.; Whitehead, S.S. Dengue virus type 3 vaccine candidates generated by introduction of deletions in the 3’ untranslated region (3’-UTR) or by exchange of the DENV-3 3’-UTR with that of DENV-4. *Vaccine* **2008**, *26*, 817–828.
35. Kieft, J.S.; Rabe, J.L.; Chapman, E.G. New hypotheses derived from the structure of a flaviviral Xrn1-resistant RNA: Conservation, folding, and host adaptation. *RNA Biol.* **2015**, *12*, 1169–1177.
36. Funk, A.; Truong, K.; Nagasaki, T.; Torres, S.; Floden, N.; Balmori Melian, E.; Edmonds, J.; Dong, H.; Shi, P.Y.; Khromykh, A.A. RNA structures required for production of subgenomic flavivirus RNA. *J. Virol.* **2010**, *84*, 11407–11417.

We would like to replace them with the correct ones here:

1. Chapman, D.A.; Darby, A.C.; Da Silva, M.; Upton, C.; Radford, A.D.; Dixon, L.K. Genomic Analysis of Highly Virulent Georgia 2007/1 Isolate of African Swine Fever Virus. *Emerg. Infect. Dis.* **2011**, *17*, 599–605, doi:10.3201/eid1704.101283.
2. Costard, S.; Wieland, B.; De Glanville, W.; Jori, F.; Rowlands, R.; Vosloo, W.; Roger, F.; Pfeiffer, D.U.; Dixon, L. African swine fever: How can global spread be prevented? *Philos. Trans. R. Soc. B Biol. Sci.* **2009**, *364*, 2683–2696, doi:10.1098/rstb.2009.0098.
3. Borca, M.V.; Ramirez-Medina, E.; Silva, E.; Vuono, E.; Rai, A.; Pruitt, S.; Holinka, L.G.; Velazquez-Salinas, L.; Zhu, J.; Gladue, D.P. Development of a Highly Effective African Swine Fever Virus Vaccine by Deletion of the I177L Gene Results in Sterile Immunity against the Current Epidemic Eurasia Strain. *J. Virol.* **2020**, *94*, doi:10.1128/jvi.02017-19.
4. O’Donnell, V.; Holinka, L.G.; Krug, P.W.; Gladue, D.P.; Carlson, J.; Sanford, B.; Alfano, M.; Kramer, E.; Lu, Z.; Arzt, J.; et al. African Swine Fever Virus Georgia 2007 with a Deletion of Virulence-Associated Gene9GL(B119L), when Administered at Low Doses, Leads to Virus Attenuation in Swine and Induces an Effective Protection against Homologous Challenge. *J. Virol.* **2015**, *89*, 8556–8566, doi:10.1128/jvi.00969-15.
5. O’Donnell, V.; Risatti, G.R.; Holinka, L.G.; Krug, P.W.; Carlson, J.; Velazquez-Salinas, L.; Azzinaro, P.A.; Gladue, D.P.; Borca, M.V. Simultaneous Deletion of the 9GL and UK Genes from the African Swine Fever Virus Georgia 2007 Isolate Offers Increased Safety and Protection against Homologous Challenge. *J. Virol.* **2016**, *91*, doi:10.1128/jvi.01760-16.
6. Reis, A.L.; Goatley, L.C.; Jabbar, T.; Sanchez-Cordon, P.J.; Netherton, C.L.; Chapman, D.A.G.; Dixon, L.K. Deletion of the African Swine Fever Virus Gene DP148R Does Not Reduce Virus Replication in Culture but Reduces Virus Virulence in Pigs and Induces High Levels of Protection against Challenge. *J. Virol.* **2017**, *91*, doi:10.1128/jvi.01428-17.
7. O’Donnell, V.; Holinka, L.G.; Gladue, D.P.; Sanford, B.; Krug, P.W.; Lu, X.; Arzt, J.; Reese, B.; Carrillo, C.; Risatti, G.R.; et al. African Swine Fever Virus Georgia Isolate Harboring Deletions of MGF360 and MGF505 Genes Is Attenuated in Swine and Confers Protection against Challenge with Virulent Parental Virus. *J. Virol.* **2015**, *89*, 6048–6056, doi:10.1128/jvi.00554-15.
8. Monteagudo, P.L.; Lacasta, A.; López, E.; Bosch, L.; Collado, J.; Pina-Pedrero, S.; Correa-Fiz, F.; Accensi, F.; Navas, M.J.; Vidal, E.; et al. BA71ΔCD2: A New Recombinant Live Attenuated African Swine Fever Virus with Cross-Protective Capabilities. *J. Virol.* **2017**, *91*, doi:10.1128/jvi.01058-17.
9. O’Donnell, V.; Holinka, L.G.; Sanford, B.; Krug, P.W.; Carlson, J.; Pacheco, J.M.; Reese, B.; Risatti, G.R.; Gladue, D.P.; Borca, M. African swine fever virus Georgia isolate harboring deletions of 9GL and MGF360/505 genes is highly attenuated in swine but does not confer protection against parental virus challenge. *Virus Res.* **2016**, *221*, 8–14, doi:10.1016/j.virusres.2016.05.014.
10. Borca, M.V.; O’Donnell, V.; Holinka, L.G.; Ramirez-Medina, E.; Clark, B.A.; Vuono, E.A.; Berggren, K.; Alfano, M.; Carey, L.B.; Richt, J.A. The L83L ORF of African swine fever virus strain Georgia encodes for a non-essential gene that interacts with the host protein IL-1β. *Virus Res.* 2018, *249*, 116–123, doi:10.1016/j.virusres.2018.03.017.
11. Ramirez-Medina, E.; Vuono, E.A.; Velazquez-Salinas, L.; Silva, E.; Rai, A.; Pruitt, S.; Berggren, K.A.; Zhu, J.; Borca, M.V.; Gladue, D. The MGF360-16R ORF of African Swine Fever Virus Strain Georgia Encodes for a Nonessential Gene That Interacts with Host Proteins SERTAD3 and SDCBP. *Viruses* **2020**, *12*, 60, doi:10.3390/v12010060.
12. Borca, M.V.; O’Donnell, V.; Holinka, L.G.; Risatti, G.R.; Ramirez-Medina, E.; Vuono, E.A.; Shi, J.; Pruitt, S.; Rai, A.; Silva, E.; et al. Deletion of CD2-like gene from the genome of African swine fever virus strain Georgia does not attenuate virulence in swine. *Sci. Rep.* **2020**, *10*, 1–8, doi:10.1038/s41598-020-57455-3.
13. Afonso, C.L.; Carrillo, C.; Zsak, L.; Rock, D.L.; Borca, M. African swine fever virus NL gene is not required for virus virulence. *J. Gen. Virol.* **1998**, *79*, 2543–2547, doi:10.1099/0022-1317-79-10-2543.
14. Sanford, B.; Holinka, L.; O’Donnell, V.; Krug, P.; Carlson, J.; Alfano, M.; Carrillo, C.; Wu, P.; Lowe, A.; Risatti, G.; et al. Deletion of the thymidine kinase gene induces complete attenuation of the Georgia isolate of African swine fever virus. *Virus Res.* **2016**, *213*, 165–171, doi:10.1016/j.virusres.2015.12.002.
15. Ramirez-Medina, E.; Vuono, E.A.; Rai, A.; Pruitt, S.; Silva, E.; Velazquez-Salinas, L.; Zhu, J.; Gladue, D.; Gladue, D. The C962R ORF of African Swine Fever Strain Georgia Is Non-Essential and Not Required for Virulence in Swine. *Viruses* **2020**, *12*, 676, doi:10.3390/v12060676.
16. Afonso, C.L.; Alcaraz, C.; Brun, A.; Sussman, M.D.; Onisk, D.V.; Escribano, J.M.; Rock, D.L. Characterization of P30, a highly antigenic membrane and secreted protein of African Swine Fever Virus. *Virology* **1992**, *189*, 368–373, doi:10.1016/0042-6822(92)90718-5.
17. Borca, M.V.; O’Donnell, V.; Holinka, L.G.; Rai, D.K.; Sanford, B.; Alfano, M.; Carlson, J.; Azzinaro, P.A.; Alonso, C.; Gladue, D. The Ep152R ORF of African swine fever virus strain Georgia encodes for an essential gene that interacts with host protein BAG6. *Virus Res.* **2016**, *223*, 181–189, doi:10.1016/j.virusres.2016.07.013.
18. Rodríguez, J.M.; García-Escudero, R.; Salas, M.L.; Andrés, G. African Swine Fever Virus Structural Protein p54 Is Essential for the Recruitment of Envelope Precursors to Assembly Sites. *J. Virol.* **2004**, *78*, 4299–1313, doi:10.1128/jvi.78.8.4299-4313.2004.
19. García-Escudero, R.; Andrés, G.; Almazán, F.; Viñuela, E. Inducible Gene Expression from African Swine Fever Virus Recombinants: Analysis of the Major Capsid Protein p72. *J. Virol.* **1998**, *72*, 3185–3195, doi:10.1128/jvi.72.4.3185-3195.1998.
20. Upton, C. Multigene families in African Swine Fever Virus. Availabe online: https://4virology.net/organisms/dsdna-viruses/asfarviridae/ (accessed on 20 July 2020).
21. Borca, M.V.; O’Donnell, V.; Holinka, L.G.; Sanford, B.; Azzinaro, P.A.; Risatti, G.R.; Gladue, D. Development of a fluorescent ASFV strain that retains the ability to cause disease in swine. *Sci. Rep.* **2017**, *7*, srep46747, doi:10.1038/srep46747.
22. Golding, J.P.; Goatley, L.; Goodbourn, S.; Dixon, L.; Taylor, G.; Netherton, C.L. Sensitivity of African swine fever virus to type I interferon is linked to genes within multigene families 360 and 505. *Virology* **2016**, *493*, 154–161, doi:10.1016/j.virol.2016.03.019.
23. Burrage, T.G.; Lu, Z.; Neilan, J.G.; Rock, D.L.; Zsak, L. African Swine Fever Virus Multigene Family 360 Genes Affect Virus Replication and Generalization of Infection in Ornithodoros porcinus Ticks. *J. Virol.* **2004**, *78*, 2445–2453, doi:10.1128/jvi.78.5.2445-2453.2004.
24. Borca, M.; Berggren, K.; Ramirez-Medina, E.; Vuono, E.; Gladue, D. CRISPR/Cas Gene Editing of a Large DNA Virus: African Swine Fever Virus. *Bio-Protocol* **2018**, *8*, 8,, doi:10.21769/bioprotoc.2978.
25. Reed, L.J.; Muench, H. A Simple Method of Estimating Fifty Percent Endpoints. *Am. J. Hyg.* **1938**, *27*, 493–497.
26. Mitchell, A.L.; Attwood, T.K.; Babbitt, P.C.; Blum, M.; Bork, P.; Bridge, A.; Brown, S.D.; Chang, H.-Y.; El-Gebali, S.; Fraser, M.I.; et al. InterPro in 2019: Improving coverage, classification and access to protein sequence annotations. *Nucleic Acids Res.* **2018**, *47*, D351–D360, doi:10.1093/nar/gky1100.
27. Alejo, A.; Matamoros, T.; Guerra, M.; Andrés, G. A Proteomic Atlas of the African Swine Fever Virus Particle. *J. Virol.* **2018**, *92*, 92, doi:10.1128/jvi.01293-18.
28. Zhu, J.J.; Ramanathan, P.; Bishop, E.A.; O’Donnell, V.; Gladue, D.; Borca, M. Mechanisms of African swine fever virus pathogenesis and immune evasion inferred from gene expression changes in infected swine macrophages. *PLoS ONE* **2019**, *14*, e0223955, doi:10.1371/journal.pone.0223955.
29. Krug, P.W.; Holinka, L.G.; O’Donnell, V.; Reese, B.; Sanford, B.; Fernandez-Sainz, I.; Gladue, D.P.; Arzt, J.; Rodriguez, L.; Risatti, G.R.; et al. The Progressive Adaptation of a Georgian Isolate of African Swine Fever Virus to Vero Cells Leads to a Gradual Attenuation of Virulence in Swine Corresponding to Major Modifications of the Viral Genome. *J. Virol.* **2014**, *89*, 2324–2332, doi:10.1128/jvi.03250-14.
30. Sánchez-Cordón, P.J.; Jabbar, T.; Berrezaie, M.; Chapman, D.; Reis, A.; Sastre, P.; Rueda, P.; Goatley, L.; Dixon, L.K. Evaluation of protection induced by immunisation of domestic pigs with deletion mutant African swine fever virus BeninΔMGF by different doses and routes. *Vaccine* **2018**, *36*, 707–715, doi:10.1016/j.vaccine.2017.12.030.
31. Neilan, J.G.; Zsak, L.; Lu, Z.; Kutish, G.F.; Afonso, C.L.; Rock, D.L. Novel Swine Virulence Determinant in the Left Variable Region of the African Swine Fever Virus Genome. *J. Virol.* **2002**, *76*, 3095–3104, doi:10.1128/jvi.76.7.3095-3104.2002.
32. Chapman, D.A.G.; Tcherepanov, V.; Upton, C.; Dixon, L. Comparison of the genome sequences of non-pathogenic and pathogenic African swine fever virus isolates. *J. Gen. Virol.* **2008**, *89*, 397–408, doi:10.1099/vir.0.83343-0.
33. De La Vega, I.; Viñuela, E.; Blasco, R. Genetic variation and multigene families in african swine fever virus. *Virology* **1990**, *179*, 234–246, doi:10.1016/0042-6822(90)90293-z.
34. Yáñez, R.J.; Rodríguez, J.M.; Nogal, M.L.; Yuste, L.; Enríquez, C.; Rodriguez, J.F.; Viñuela, E. Analysis of the Complete Nucleotide Sequence of African Swine Fever Virus. *Virology* **1995**, *208*, 249–278, doi:10.1006/viro.1995.1149.
35. Pires, S.; Ribeiro, G.; Costa, J.V. Sequence and Organization of the Left Multigene Family 110 Region of the Vero-adapted L60V Strain of African Swine Fever Virus. *Virus Genes* **1997**, *15*, 271–274, doi:10.1023/a:1007992806818.
36. Zani, L.; Forth, J.H.; Forth, L.; Nurmoja, I.; Leidenberger, S.; Henke, J.; Carlson, J.; Breidenstein, C.; Viltrop, A.; Höper, D.; et al. Deletion at the 5’-end of Estonian ASFV strains associated with an attenuated phenotype. *Sci. Rep.* **2018**, *8*, 6510, doi:10.1038/s41598-018-24740-1.

The published version will be updated on the article webpage, with a reference to this erratum.
